# Supplementary material for: Causal Relationship between Aging and Anorexia Nervosa: A White-Matter-Microstructure-Mediated Mendelian Randomization Analysis
Source: Biomedicines. 2024 Aug 16;12(8):1874. doi: 10.3390/biomedicines12081874 (PMC11351342; doi:10.3390/biomedicines12081874)
Supplement: Supplementary file 1 [file biomedicines-12-01874-s001.zip › Supplementary Figure/Figure S4/Singe SNP plot of MR analysis for TL to ubm-b-1494.pdf]

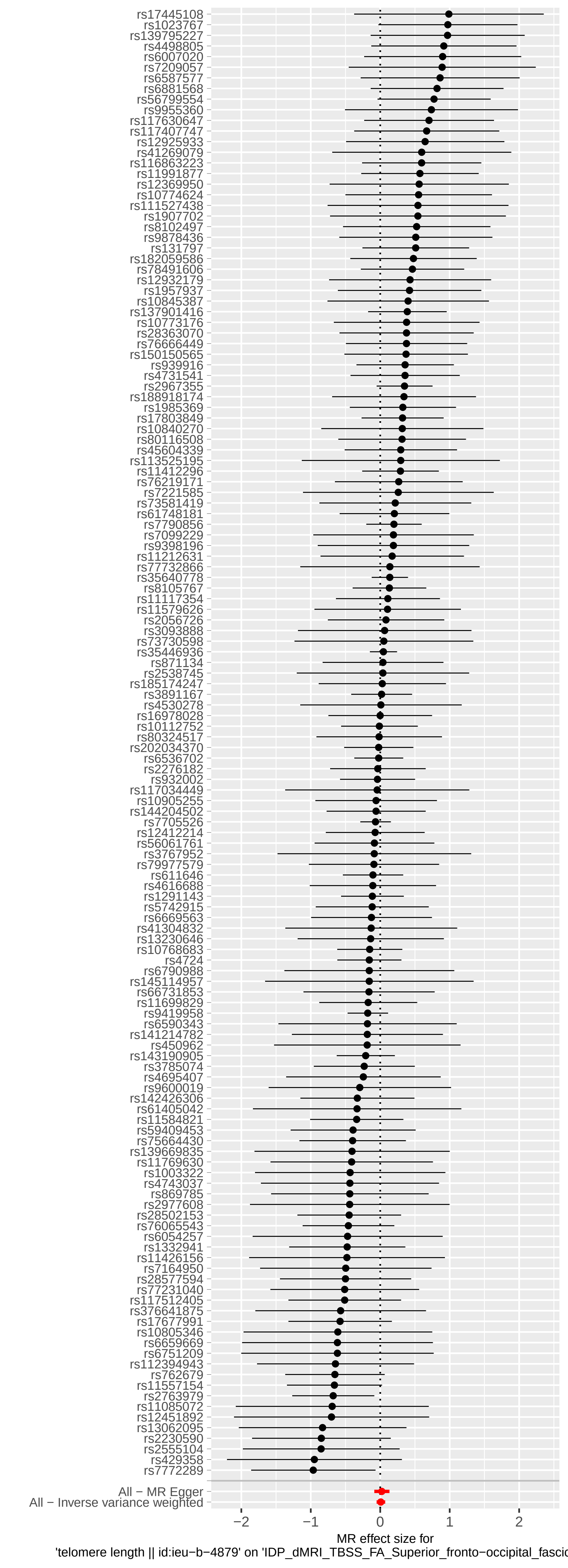

rs17445108
rs1023767
rs139795227
rs4498805
rs6007020
rs7209057
rs6587577
rs6881568
rs56799554
rs9955360
rs117630647
rs117407747
rs12925933
rs41269079
rs116863223
rs11991877
rs12369950
rs10774624
rs111527438
rs1907702
rs8102497
rs9878436
rs131797
rs182059586
rs78491606
rs12932179
rs1957937
rs10845387
rs137901416
rs10773176
rs28363070
rs76666449
rs150150565
rs939916
rs4731541
rs2967355
rs188918174
rs1985369
rs17803849
rs10840270
rs80116508
rs45604339
rs113525195
rs11412296
rs76219171
rs7221585
rs73581419
rs61748181
rs7790856
rs7099229
rs9398196
rs11212631
rs77732866
rs35640778
rs8105767
rs11117354
rs11579626
rs2056726
rs3093888
rs73730598
rs35446936
rs871134
rs2538745
rs185174247
rs3891167
rs4530278
rs16978028
rs10112752
rs80324517
rs202034370
rs6536702
rs2276182
rs932002
rs117034449
rs10905255
rs144204502
rs7705526
rs12412214
rs56061761
rs3767952
rs79977579
rs611646
rs4616688
rs1291143
rs5742915
rs6669563
rs41304832
rs13230646
rs10768683
rs4724
rs6790988
rs145114957
rs66731853
rs11699829
rs9419958
rs6590343
rs141214782
rs450962
rs143190905
rs3785074
rs4695407
rs9600019
rs142426306
rs61405042
rs11584821
rs59409453
rs75664430
rs139669835
rs11769630
rs1003322
rs4743037
rs869785
rs2977608
rs28502153
rs76065543
rs6054257
rs1332941
rs11426156
rs7164950
rs28577594
rs77231040
rs117512405
rs376641875
rs17677991
rs10805346
rs6659669
rs6751209
rs112394943
rs762679
rs11557154
rs2763979
rs11085072
rs12451892
rs13062095
rs2230590
rs2555104
rs429358
rs7772289
All − MR Egger
All − Inverse variance weighted
−2
−1
0
1
2
MR effect size for
'telomere length || id:ieu−b−4879' on 'IDP_dMRI_TBSS_FA_Superior_fronto−occipital_fascic
